# Vitamin D_3_ Reshapes Gut Microbiota and Metabolite Profiles in a Rat Model of Inflammation-Induced Myopia

**DOI:** 10.3390/biom16070939

**Published:** 2026-06-24

**Authors:** Yung-Lan Chou, Hui-Ju Lin, Yu-An Hsu, En-Shyh Lin, Chih-Sheng Chen, Peng-Tai Tien, Jamie Jiin-Yi Chen, Ming-Yen Wu, Chun-Yu Chuang, Lei Wan

**Affiliations:** 1 Department of Biomedical Engineering and Environmental Sciences, National Tsing Hua University, Hsinchu 300044, Taiwan; 2 School of Chinese Medicine, China Medical University, Taichung 404328, Taiwan; 3 Eye Center, China Medical University Hospital, Taichung 404327, Taiwan; 4 Department of Chemistry, National Central University, Taoyuan 320317, Taiwan; 5 Department of Beauty Science, National Taichung University of Science and Technology, Taichung 404336, Taiwan; 6 Division of Chinese Medicine, Asia University Hospital, Taichung 413505, Taiwan; 7 Department of Food Nutrition and Health Biotechnology, Asia University, Taichung 413305, Taiwan; 8 School of Medicine, China Medical University, Taichung 404328, Taiwan; 9 Office of Research and Development, Asia University, Taichung 413305, Taiwan; 10 Department of Obstetrics and Gynecology, China Medical University Hospital, Taichung 404327, Taiwan

**Keywords:** myopia, vitamin D_3_, gut microbiota, inflammation, imidazole, bile acid

## Abstract

Myopia is increasingly recognized as an inflammatory ocular disease. Vitamin D_3_ is a potential modulator of the gut–eye axis, but its role in inflammation-induced myopia remains unclear. This study investigated whether vitamin D_3_ supplementation attenuates myopia progression by regulating retinal inflammation, gut microbiota composition, and microbiota-derived metabolites in a TGF-β2–induced myopia model. Three-week-old Brown Norway rats received weekly periocular TGF-β2 injections with or without daily oral vitamin D_3_, and myopia development was evaluated on days 1 and 21 by axial length and refractive error. Cecal contents were analyzed for α- and β-diversity and taxonomic differences, and day-21 serum underwent untargeted metabolomic profiling of microbiota-derived metabolites, including bile acids and imidazole derivatives; Spearman correlation linked microbial or metabolic alterations with myopia progression. TGF-β2 induced axial elongation, myopic refractive shifts, and upregulated retinal pro-inflammatory cytokines (p-NFκB, IL-1β, TNF-α), while vitamin D_3_ supplementation markedly attenuated myopia progression and retinal inflammation. Cecal α-diversity did not differ among control, vitamin D_3_, TGF-β2, and TGF-β2+vitamin D_3_ groups, but vitamin D_3_ significantly reshaped β-diversity and reduced the Firmicutes/Bacteroidota ratio. Distinct metabolite profiles were observed, with the vitamin D_3_ group showing reduced hyodeoxycholic acid and elevated imidazole derivatives (imidazolepropionic and methylimidazoleacetic acids). Vitamin D_3_ supplementation attenuated myopia progression by reducing retinal inflammation and concurrently reshaping the gut microbiome and its metabolites compared to the control and myopic groups. These results underscore the potential of vitamin D_3_ to modulate the gut–retina axis as a nutritional approach for mitigating myopia development.

## 1. Introduction

Myopia, commonly referred to as near-sightedness, is recognized as an epidemic due to its rapidly increasing prevalence [1]. Statistics show that up to 90% of the Asian population is affected by myopia; it is estimated that by 2050, approximately 50% of the global population will have developed myopia [2,3]. Although the exact mechanisms underlying myopia remain unclear, it has been increasingly recognized as an inflammatory ocular condition [4]. Previously, inflammation has been implicated as a key contributor to the pathogenesis and progression of myopia. Inflammatory ocular conditions, chronic exposure to air pollutants, and local administration of pro-inflammatory cytokines were all associated with axial elongation and refractive changes, supporting a causal link between inflammation and myopic remodeling [5,6]. Ocular inflammatory diseases promote myopia development and increase the retinal expression of IL-6, NF-κB, c-Fos, and TNF-α [7]. Notably, pharmacological intervention with anti-inflammatory agents such as resveratrol and diacerein can effectively reduce retinal inflammatory signaling and attenuate myopia progression in experimental models [6,8]. These findings suggest that inflammation is crucial for myopia development, underscoring the therapeutic potential of targeting inflammatory pathways to prevent or decelerate myopia progression.

The gut microbiota is a complex and dynamic microbial ecosystem that plays a central role in host physiology, including nutrient metabolism, epithelial barrier maintenance, and immune modulation [9]. Dysbiosis of the gut microbiota can disrupt intestinal barrier integrity and promote the translocation of microbial products, leading to systemic low-grade inflammation and immune dysregulation [10,11]. Emerging evidence suggests that the gut–eye axis plays an important role in the pathogenesis of myopia. A Mendelian randomization study identified that the class Betaproteobacteria and the order Burkholderiales exhibit an increased myopia risk, highlighting a causal link between gut microbial composition and refractive development [12]. Furthermore, a microbiota-derived metabolite, indole-3-acetic acid (3-IAA), has been shown to prevent high myopia progression by maintaining scleral integrity through the promotion of type I collagen synthesis [13]. In our previous work, we demonstrated that the prebiotic *Lactiplantibacillus plantarum* EP21 and its membrane vesicles attenuated myopia development by alleviating retinal inflammation [14]. Collectively, these findings underscore the research importance and therapeutic potential of the gut–eye axis in the progression of myopia.

Vitamin D_3_, a prevalent nutritional supplement, has been recognized as an anti-inflammatory agent and is potentially related to myopia development [15,16]. Numerous investigations have indicated that the incidence of myopia is exacerbated by diminished serum concentrations of vitamin D_3_ [17,18]. In contrast, supplementation with oral vitamin D can augment blood levels of 25(OH)D and marginally reduce axial length in myopic individuals aged 15 to 25 [19]. A separate investigation revealed that calcipotriol treatment mitigates the progression of myopia by enhancing scleral COL1A1 expression via a scleral vitamin D receptor (VDR)-dependent mechanism in a form-deprivation myopia (FDM) animal model [20]. Moreover, vitamin D_3_ has been linked to intestinal barrier regulation, influencing the immune system and gut microbiota. Lower serum vitamin D levels are associated with various intestinal diseases, suggesting that vitamin D helps maintain a healthy gut microbiome [12]. A growing body of work has described a gut microbiota–bile acid–vitamin D axis wherein VDR signaling influences bile acid synthesis and transport, while bile acids and microbiota modulate vitamin D metabolism and immune homeostasis [21]. These interactions suggest that vitamin D_3_ supplementation may reshape gut microbial ecology and the production of bioactive metabolites, thereby indirectly affecting systemic and ocular inflammation [22].

These observations suggest a potential vitamin D_3_–gut microbiota–metabolite axis that may modulate ocular inflammation and myopia progression; however, the involvement of specific bile acids and imidazole metabolites remains poorly defined. In this study, we aimed to elucidate the relationship between vitamin D_3_ supplementation and myopia development via gut microbiota and metabolic alterations in an inflammation-induced myopia animal model.

## 2. Materials and Methods

### 2.1. Animal Model

Three-week-old male Brown-Norway rats from the National Laboratory Animal Center (Taipei, Taiwan). The rats were housed under a 12 h light/12 h dark cycle, with the light phase provided by incandescent lamps. They were fed Laboratory Rodent Diet 5001 (LabDiet, Richmond, IN, USA), which contains 0.95% calcium and 4.6 IU/g cholecalciferol. All experimental procedures were approved by the Institutional Animal Care and Use Committee of China Medical University (CMUIACUC-2023-295) and were in accordance with the guidelines for the Use of Animals in Ophthalmic and Vision Research.

The animals were randomly allocated into four groups: the control group (*n* = 6), the vitamin D_3_ group (*n* = 9), the TGF-β2 group (*n* = 9), and the TGF-β2+vitamin D_3_ group (*n* = 9). In the control group, animals received a daily oral gavage of 100 μL castor oil. Rats in the vitamin D_3_ group received 1000 IU/kg vitamin D_3_ (11792, Cayman, Ann Arbor, MI, USA) by oral gavage, corresponding to 1 IU vitamin D_3_ per gram of body weight, dissolved in 100 μL castor oil. Animals were administered a weekly eyelid injection of TGF-β2 (250 ng/mL) (100-35B, PeproTech, Cranbury, NJ, USA) to induce myopia in the TGF-β2 group, along with a daily oral dose of 100 μL castor oil. In the TGF-β2+vitamin D_3_ group, the animals’ eyes were injected with 250 ng/mL TGF-β2 once a week, and vitamin D_3_ was administered daily. Rats were weighed on Days 1, 7, 14, and 21, and the vitamin D_3_ dose was adjusted weekly to ensure that each animal consistently received 1000 IU/kg/day. The experiment was conducted over a total of 21 days, with axial length and refractive error measurements taken at both the start and the end of the study.

### 2.2. Sample Collection

At the beginning of the experiment, blood for serum collection was obtained via facial vein puncture. At the end of the study, animals were placed under deep isoflurane anesthesia, and intracardiac blood collection was performed only after complete loss of corneal and toe-pinch withdrawal reflexes was confirmed. Following euthanasia, eye tissues and cecal contents were harvested for further analyses. Serum was isolated by centrifugation and immediately stored at −80 °C for subsequent 25-hydroxyvitamin D_3_ and metabolomics analyses. Eyes were fixed and embedded in paraffin for immunofluorescence staining. Fecal samples were collected from the cecum and stored at −80 °C for microbiota analysis.

### 2.3. Immunofluorescence Staining

The eye tissues were embedded in paraffin and sectioned at a thickness of 3 μm. After rehydration, the sections were subjected to antigen retrieval for 20 min using Epitope Retrieval Solution (Leica Biosystems, Buffalo Grove, IL, USA). Subsequently, the sections were blocked with 10% goat serum for 30 min at room temperature and then incubated overnight at 4 °C with specific primary antibodies against TGF-β2 (GTX132546, Genetex, Irvine, CA, USA), MMP-2 (ab97779, Abcam, Cambridge, UK), p-NFκB (3033S, Cell signaling, Danvers, MA, USA), TNF-α (BS1857, Bloomington, MN, USA), and IL-1β (ab9722, Abcam, Cambridge, UK). The following day, the samples were incubated with a Cy3-conjugated secondary antibody (111-165-003, Jackson ImmunoResearch, West Grove, PA, USA) for 1 h at 25 °C, followed by a 5 min incubation with DAPI at the same temperature. Finally, the samples were mounted with coverslips using FluoreGuard mounting medium (FMM030, ScyTek Laboratories, Logan, UT, USA).

### 2.4. Serum 25-Hydroxyvitamin D_3_ Analysis

Serum 25-hydroxyvitamin D_3_ (25(OH)D_3_) levels of day 1 and day 21 were measured using a commercial enzyme-linked immunosorbent assay (ELISA) kit (Rat 25-HO-D ELISA Kit, EK0843; ELK Biotechnology, Sugar Land, TX, USA), following the manufacturer’s instructions (*n* = 4).

### 2.5. Gut Microbiota Analysis

Total DNA was extracted from cecal fecal samples using the ZymoBIOMICS™ DNA Miniprep Kit (D4300, Zymo Research, Irvine, CA, USA). The V3–V4 region of the 16S rRNA gene was amplified using specific primers (forward: 5′-ACTCCTACGGGAGGCAGCAG-3′ and reverse: 5′-GGACTACHVGGGTWTCTAAT-3′). Sequencing of the amplicon pools was performed on the Illumina MiSeq™ platform (Illumina, San Diego, CA, USA). Data were analyzed using QIIME (1.9.1). Raw reads were demultiplexed using the q2-demux plugin and then processed for denoising and quality filtering with the q2-dada2 plugin, resulting in an amplicon sequence variant (ASV) table.

Alpha diversity was evaluated using the Chao1 and Shannon indices based on OTU analysis results. Beta diversity was assessed by Principal Coordinates Analysis (PCoA) based on Bray–Curtis distance matrices and non-metric multidimensional scaling (NMDS) analysis based on the weighted UniFrac distance matrix. Both alpha diversity and beta diversity were analyzed using R software (version 3.3.1). To identify differentially abundant taxa among groups, Linear Discriminant Analysis Effect Size (LEfSe) analysis was performed with an LDA value greater than 3. Additionally, Statistical Analysis of Metagenomic Profiles (STAMP) was used for statistical comparisons among the groups. Microbial functional prediction was performed using Phylogenetic Investigation of Communities by Reconstruction of Unobserved States (PICRUSt) analysis based on 16S rRNA gene sequencing data (PICRUSt, version 1.0.0). The correlation between myopia and gut microbiota was assessed using Spearman’s correlation coefficient analysis in R software, based on ASV abundance, species abundance, and measurements of axial length or refractive error.

### 2.6. Untargeted Metabolomic Analysis

An aliquot of 200 μL of serum (*n* = 4 for each group) was added to acetone in a 1:4 volume ratio to precipitate and remove the protein pellet. The supernatant was then collected and vacuum-dried. The dried supernatant was then re-dissolved in 200 μL of 5% methanol/0.1% formic acid, and 5 μL of the treated serum sample was subjected to LC-MS analysis.

The chromatographic separation was performed at a flow rate of 250 μL/min using solvent A (0.1% formic acid in water) and solvent B (100% acetonitrile). The gradient elution started with 1% solvent B for 1 min, ramped to 40% over the next 14 min, followed by a further increase to 80% within 3 min. This was maintained for 3 min, then returned to 1% and held for equilibration over 5 min, resulting in a total run time of 25 min. The chromatographic column was a 3 μm 100 Å ODS, 2.1 × 150 mm (Atlantis T3, Waters, Milford, MA, USA) equipped with a Q-TRAP 6500+ system (AB Sciex, Framingham, MA, USA). The column operated at a temperature of 35 °C.

In negative ion mode, the curtain gas (CUR) was set to 25 psi, the collision gas (CAD) to 12, and the ion spray voltage (IS) to −4500 V. In positive ion mode, CUR was maintained at 25 psi, CAD at 10, and IS at +5500 V. For both modes, the source temperature was set at 500 °C, with gas 1 (GS1) and gas 2 (GS2) set to 50 and 60 psi, respectively. Multiple reaction monitoring (MRM) mode was employed for compound detection. For each target metabolite, the precursor ion (*m*/*z*), fragment ion (*m*/*z*), and retention time were individually defined. Compound intensity profiles were generated by integrating the analyst peak areas using OS software (AB Sciex).

### 2.7. Statistical Analysis

Statistical analysis of axial length and refractive error was expressed as mean ± standard deviation (SD) from at least three independent experiments. One-way ANOVA was conducted to compare multiple groups using GraphPad Prism software (version 10, GraphPad Software, San Diego, CA, USA). Between-group differences in α-diversity were assessed using independent *t*-tests. Between-group differences in β-diversity were assessed using permutational multivariate analysis of variance (PERMANOVA) based on weighted UniFrac distance matrices; resulting *p*-values were adjusted for multiple testing using the Benjamini–Hochberg false discovery rate (FDR) procedure. The stack bar of relative abundance at the phylum level and PICRUSt were plotted using https://www.bioinformatics.com.cn (last accessed 10 December 2024), an online platform for data analysis and visualization. Differential metabolite expression among groups was visualized in a heatmap based on z-score values, while log2 fold change (log2FC) and *p*-values were also calculated. Correlation analyses between myopia development and microbial community composition or metabolite levels were performed using Spearman’s correlation coefficient in R software, based on changes in axial length, diopter, operational taxonomic unit (OTU) abundance, species abundance, and metabolite data. Differences were considered statistically significant at *p* < 0.05.

## 3. Results

### 3.1. Vitamin D_3_ Attenuates TGF-β2–Induced Myopia and Inflammation in the Eye

Previously, we demonstrated that TGF-β2 promotes ocular inflammation, contributing to myopia progression [23,24]. Experimental myopia was induced in rats via weekly eyelid injections of TGF-β2 over 3 weeks, with concurrent oral administration of vitamin D_3_ being implemented as a therapeutic intervention. The comparison of systematic measurements of axial length and refractive error on Day 1 and 21 was used to assess myopia development.

**Figure 1:**
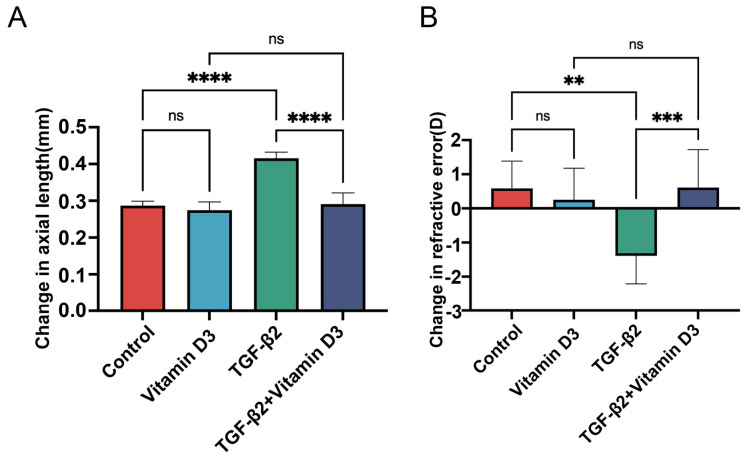
Vitamin D_3_ mitigates TGF-β2-induced myopia progression. (**A**) Axial length elongation induced by TGF-β2 eyelid injection significantly reduces with vitamin D_3_ supplementation. (**B**) Vitamin D_3_ intake inhibits the marked myopic refractive shift induced by TGF-β2. Changes in axial length (mm) and refractive error (diopters) were calculated as the difference between day 1 and 21 values in each animal. Group comparisons were analyzed using one-way ANOVA followed by post hoc multiple comparisons (*n* = 6 in the control group, *n* = 9 in the vitamin D_3_, TGF-β2, and TGF-β2+vitamin D_3_ groups). Statistical significance: ** *p* < 0.01, *** *p* < 0.0005, **** *p* < 0.0001, ns: not significant.

After 3 weeks, the TGF-β2 group displayed a significantly greater axial length (0.42 ± 0.02 mm) compared to the control (0.29 ± 0.01 mm, *p* < 0.05). In contrast, oral vitamin D_3_ supplementation effectively suppressed axial elongation, resulting in reduced axial lengths for the vitamin D_3_ (0.27 ± 0.02 mm) and TGF-β2+vitamin D_3_ groups (0.29 ± 0.03 mm) that were indistinguishable from the control group (Figure 1A). The TGF-β2 group also exhibited a markedly decreased myopic refractive error (−1.39 ± 0.82 D) compared to the control group (0.58 ± 0.80 D, *p* < 0.05). In comparison, vitamin D_3_ intervention normalized refractive indices in the vitamin D_3_ (0.25 ± 0.93 D) and TGF-β2+vitamin D_3_ groups (0.61 ± 1.11 D), with values similar to those in the control group (Figure 1B).

**Figure 2:**
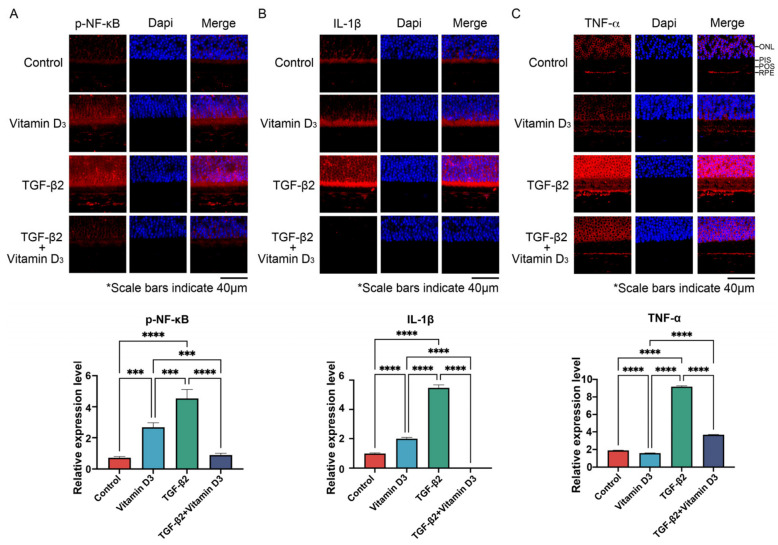
Vitamin D_3_ attenuates TGF-β2-induced retinal inflammation. Immunofluorescence staining shows retinal expression of the pro-inflammatory markers ((**A**) phosphorylated NF-κB (p-NF-κB), (**B**) IL-1β, and (**C**) TNF-α) in control, vitamin D_3_, TGF-β2, and TGF-β2+vitamin D_3_ groups, while revealing the relative fluorescence intensity. Differences among groups were evaluated using one-way ANOVA followed by post hoc multiple comparisons. Statistical significance: *** *p* < 0.0005, **** *p* < 0.0001, ns: not significant. ONL, outer nuclear layer; PIS, photoreceptor inner segments; POS, photoreceptor outer segments; RPE, retinal pigment epithelium.

We previously identified TGF-β2 and MMP-2 as markers associated with myopia linked to retinal extracellular matrix remodeling [23]. In the current study, our results showed that vitamin D_3_ administration significantly suppressed the upregulation of TGF-β2 and MMP-2 (Supplementary Figure S1) induced by TGF-β2 after 3 weeks. Additionally, elevated levels of inflammatory mediators, including p-NFκB, IL-1β, and TNF-α, were observed in the TGF-β2 group, which were reversed after vitamin D_3_ supplementation (Figure 2).

These findings suggest that vitamin D_3_ confers protective effects against myopia progression by concurrently suppressing axial elongation and refractive shifts associated with the attenuation of ocular inflammation.

### 3.2. α-Diversity and the Change in Phylum Structure

**Figure 3:**
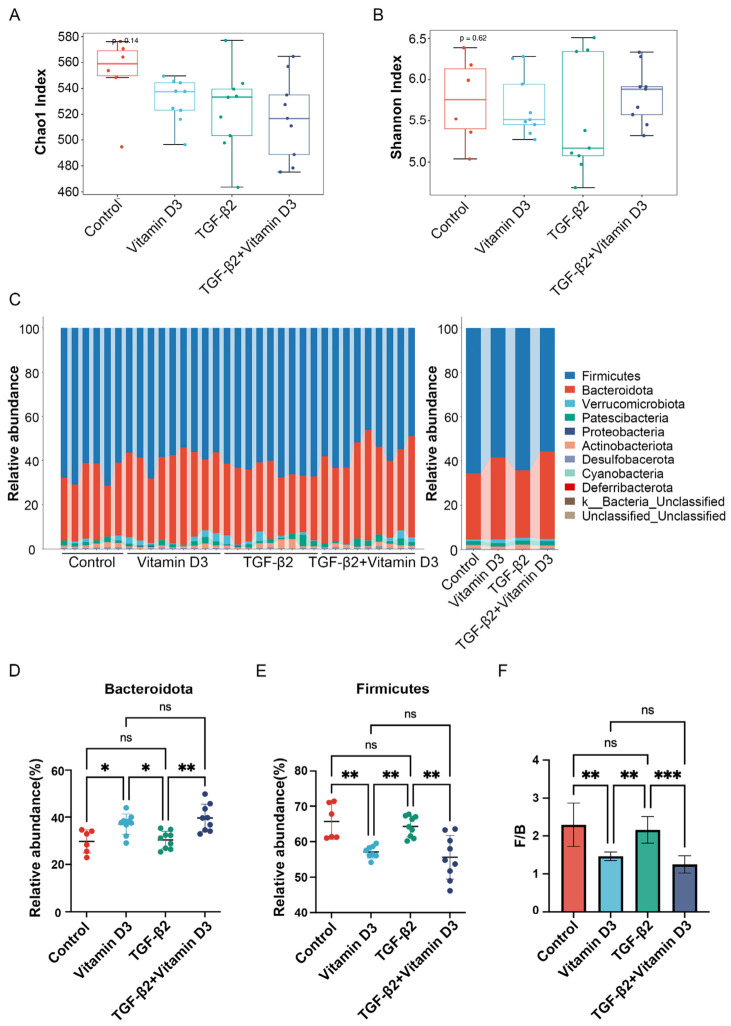
Alterations in gut microbial α-diversity and community composition at the phylum level during myopia development. (**A**,**B**) α-diversity of cecal microbiota in control (*n* = 6), vitamin D_3_ (*n* = 9), TGF-β2 (*n* = 9), and TGF-β2+vitamin D_3_ (*n* = 9) groups assessed using Chao1 index (**A**) and Shannon index (**B**). (**C**) Stacked bar plots showing the relative abundance of major bacterial phyla in each group. (**D**,**E**) Effects of vitamin D_3_ and TGF-β2 on the relative abundances of Bacteroidota (**D**) and Firmicutes (**E**), which have been analyzed using one-way ANOVA followed by post hoc multiple comparisons. (**F**) Firmicutes-to-Bacteroidota (F/B) ratio in each group, highlighting changes in the vitamin D_3_ and TGF-β2+vitamin D_3_ groups compared to the control and TGF-β2 groups. Statistical significance: * *p* < 0.05, ** *p* < 0.01, *** *p* < 0.0005; ns: not significant.

The gut microbiota composition was assessed using 16S rRNA sequencing. Both the Chao1 (Figure 3A) and Shannon indexes (Figure 3B) demonstrated no significant differences among the control, vitamin D_3_, TGF-β2, and TGF-β2+vitamin D_3_ groups, indicating that α-diversity remained stable regardless of myopia induction or vitamin D_3_ supplementation (Table 1).

Analysis at the phylum level revealed that vitamin D_3_ administration altered the gut microbial composition (Figure 3C). Compared to the control (29.74%) and TGF-β2 groups (30.36%), the relative abundance of Bacteroidota increased in the vitamin D_3_ (37.07%) and TGF-β2+vitamin D_3_ groups (39.64%) (Figure 3D). In parallel, Firmicutes’ abundance was reduced in the vitamin D_3_ (58.31%) and TGF-β2+vitamin D_3_ groups (55.61%) compared to the control (65.67%) and TGF-β2 groups (64.28%) (Figure 3E). These results suggest that vitamin D_3_ supplementation may reshape the gut microbiota composition by decreasing the Firmicutes-to-Bacteroidota ratio (F/B) (Figure 3F).

### 3.3. The Change in the Gut Microbiome Is Associated with Myopia Development

**Figure 4:**
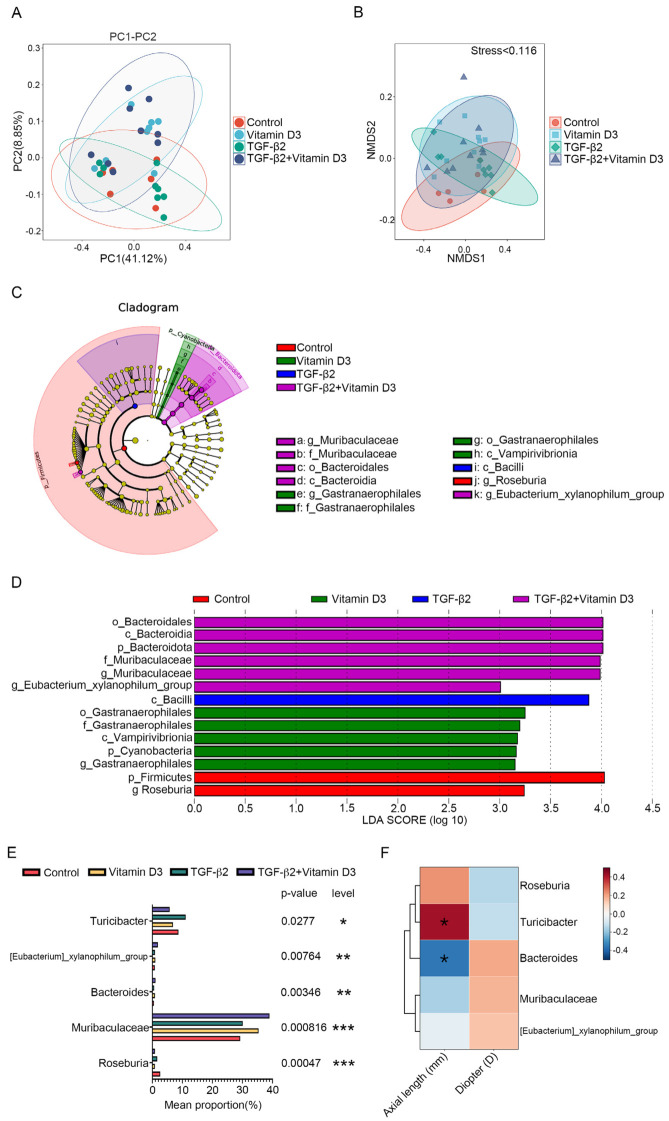
Vitamin D_3_-associated shifts in gut microbiota composition related to myopia development. (**A**,**B**) β-diversity of cecal microbiota in control (*n* = 6), vitamin D_3_ (*n* = 9), TGF-β2 (*n* = 9), and TGF-β2+vitamin D_3_ (*n* = 9) groups, which were visualized using PCoA (**A**) and NMDS (**B**) based on weighted UniFrac distance metrics. (**C**) Cladogram generated by LEfSE showing differentially enriched taxa (LDA > 3.0) among groups, with red, green, blue, and purple highlighting taxa enriched in control, vitamin D_3_, TGF-β2, and TGF-β2+vitamin D_3_ groups, respectively. (**D**) Histogram of LDA scores (log10) for taxa with significant differential abundance (LDA > 3.0). (**E**) Differentially abundant genera identified by STAMP analysis across the four groups, with group differences evaluated using one-way ANOVA followed by post hoc multiple comparisons. (**F**) Spearman’s correlation analysis between differential bacteria identified from STAMP analysis and axial length or diopter. * *p* < 0.05, ** *p* < 0.01, *** *p* < 0.0005 indicates statistical significance.

To assess changes in gut microbiota composition in myopic animals and following vitamin D_3_ administration, β-diversity was evaluated using PCoA and NMDS based on weighted UniFrac distance matrices. PERMANOVA showed that β-diversity in the vitamin D_3_ and TGF-β2+vitamin D_3_ group differed significantly from the control and TGF-β2 groups (*p* = 0.032 and *p* = 0.013 for vitamin D_3_ and TGF-β2+vitamin D_3_ group, respectively, compared to control; *p* = 0.032 and *p* = 0.007 compared to the TGF-β2 group), whereas the differences between the control and TGF-β2, and between vitamin D_3_ and TGF-β2+vitamin D_3_, were insignificant after correction for multiple testing (Figure 4A,B and Table 2).

Distinct bacterial taxa demonstrating significant differential abundance between groups were identified using LEfSe (LDA score ≥ 3, Figure 4C). The vitamin D_3_ and TGF-β2+vitamin D_3_ supplementation groups exhibited a greater number of significantly enriched taxa. Notably, the phylum Cyanobacteria, which has been reported to modulate the immune response [24], was enriched in the vitamin D_3_ group. In the TGF-β2+vitamin D_3_ group, Bacteroidales [25], *Muribaculaceae* [26], and *Eubacterium_xylanophilum_group* [27] showed significant enrichment (Figure 4D). STAMP analysis was employed for further characterization at the genus level, revealing that the two vitamin D_3_-supplemented groups exhibited highly similar microbial profiles, which differed significantly from the control and TGF-β2-only groups (Figure 4E). Specifically, *Eubacterium_xylanophilum_group*, *Bacteroides*, and *Muribaculaceae* abundance was enhanced following vitamin D_3_ treatment, whereas that of *Turicibacter* and *Roseburia* was reduced.

To evaluate the link between microbial alteration and myopia development, Spearman correlation analysis was performed. *Turicibacter* was positively correlated with axial elongation (R = 0.422, *p* = 0.015), while Bacteroides was significantly correlated with shorter axial length (R = −0.356, *p* = 0.042; Figure 4F). These findings suggest that vitamin D_3_ supplementation results in distinct changes in gut microbial composition, which are associated with the modulation of myopia progression.

### 3.4. Alternation of Bile Acids and Imidazole Derivatives May Be Involved in the Suppression of Vitamin D_3_ During Myopia Development

**Figure 5:**
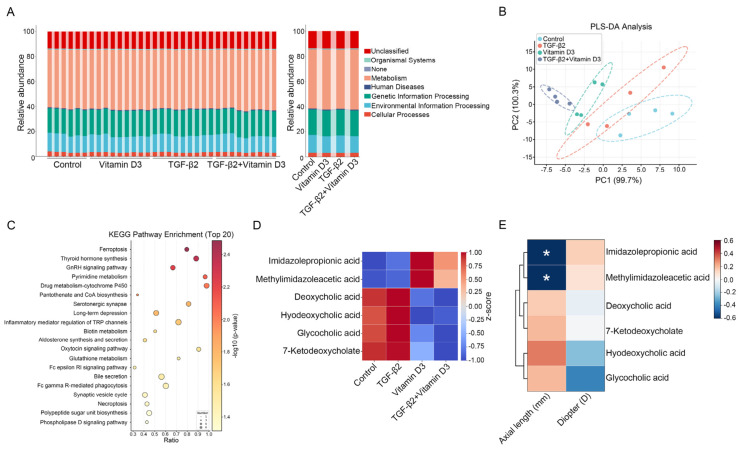
Vitamin D_3_ modulates gut microbiota–derived metabolites associated with myopia development. (**A**) Stacked bar plots of predicted microbiota functions (KEGG level 2) in control (*n* = 6), vitamin D_3_ (*n* = 9), TGF-β2 (*n* = 9), and TGF-β2+vitamin D_3_ (*n* = 9) groups. (**B**) Partial Least Squares Discriminant Analysis (PLS-DA) score plot showing separation of serum metabolite profiles among control (*n* = 4), vitamin D_3_ (*n* = 4), TGF-β2 (*n* = 4), and TGF-β2+vitamin D_3_ (*n* = 4) groups. (**C**) Bubble plot of the top 20 KEGG pathways enriched in differential metabolites; dot size represents the number of metabolites per pathway, and dot color reflects significance (−log10 *p*-value). (**D**) Heatmap (z-score) illustrating group-wise differences in bile acids and imidazole derivatives. (**E**) Spearman’s correlation heatmap between metabolites and myopia parameters (axial length and diopter); * *p* < 0.05 indicates statistical significance.

Alterations in gut microbiome composition have been shown to influence diverse host biological functions [28]. To elucidate the functional potential of the gut microbiota, predicted bacterial functions were analyzed using PICRUSt based on the Kyoto Encyclopedia of Genes and Genomes (KEGG) database. Among the four experimental groups, metabolic functions exhibited the highest relative abundance, accounting for 47.00, 47.72, 47.15, and 47.88% in the control, vitamin D_3_, TGF-β2, and TGF-β2+vitamin D_3_ groups, respectively (Figure 5A). To further investigate the impact of gut microbiota shifts, plasma metabolite profiling was performed by PLS-DA analysis, revealing distinct metabolic phenotypes across the groups (Figure 5B). Pathway enrichment analysis based on the KEGG database identified several inflammation-related pathways that were differentially regulated, including drug metabolism–cytochrome P450, inflammatory mediator regulation of TRP channels, Fc epsilon RI signaling pathway, bile secretion, Fc gamma R-mediated phagocytosis, necroptosis, and glutathione metabolism (Figure 5C). Notably, metabolites associated with the bile secretion and drug Metabolism–Cytochrome P450 pathways—such as bile acid and imidazole derivatives—have been implicated in ocular diseases [29,30,31].

The expression levels of bile acid and imidazole derivatives showed similar trends in the vitamin D_3_-treated groups (vitamin D_3_ and TGF-β2+vitamin D_3_) with the exhibited patterns being more closely aligned with each other than with the control or TGF-β2 groups (Figure 5D). Imidazole derivatives—such as imidazolepropionic acid (ImP) and methylimidazoleacetic acid (MIAA)—were significantly upregulated in the vitamin D_3_ group compared to the control and TGF-β2 groups (*p* = 0.006 and *p* = 0.011 for ImP and MIAA, respectively, compared to control; *p* = 0.008 and *p* = 0.016 compared to the TGF-β2 group). In contrast, hyodeoxycholic acid (HDCA) showed a significant decrease in the vitamin D_3_ group compared to the TGF-β2 group (*p* = 0.001). Other bile acid derivatives—including deoxycholic acid (DCA), HDCA, glycocholic acid (GCA), and 7-ketodeoxycholate—showed a decreasing trend in both vitamin D_3_-administered groups compared to the control and TGF-β2 groups, but the differences were not statistically significant (Supplementary Tables S1 and S2). To clarify the relationship between metabolite dynamics and myopia suppression induced by vitamin D_3_, Spearman correlation analysis was performed to examine the association between altered metabolite levels and measures of myopia development. Remarkably, ImP and MIAA exhibited significantly negative relationships with the axial length elongation (Figure 5E). These findings suggest that vitamin D_3_ administration can modulate gut microbiota composition and metabolic output—specifically, bile acid and imidazole derivatives—which may contribute to the suppression of retinal inflammation and inhibition of myopia progression.

## 4. Discussion

Recently, accumulating evidence has highlighted the gut–eye axis as a critical pathway through which the gut microbiota can affect ocular health by modulating immune responses and inflammation, key processes in the pathogenesis of eye disease. Nevertheless, whether vitamin D_3_–induced alterations in gut microbiota contribute to myopia development remains poorly understood. In this study, we investigated whether vitamin D_3_ attenuates inflammation-induced myopia and examined whether these effects are associated with vitamin D_3_-dependent changes in the gut microbiome composition.

Previously, ocular inflammation has been shown to promote myopia development, and atropine, a drug widely used clinically for myopia control, has been found to reduce inflammatory mediators in the retina [7]. Anti-inflammatory agents, such as diacerein and resveratrol, likewise suppressed monocular form-deprivation myopia by attenuating retinal inflammation in animal models [6,8]. Together, these findings highlight a strong association between inflammatory pathways and myopia progression. In the present study, we demonstrated the anti-inflammatory effect of vitamin D_3_ and its ability to decelerate myopia progression in an inflammation-induced myopia animal model, suggesting that vitamin D_3_ may inhibit myopia development by attenuating retinal inflammation.

Bacteroidota and Firmicutes are the two dominant bacterial phyla in the mammalian gut, each contributing in distinct ways to host immune regulation [32]. Recently, shifts in the F/B ratio have attracted increasing interest as a potential marker of disease-associated dysbiosis. In inflammatory bowel disease and certain refractory rheumatoid arthritis cohorts, markedly reduced F/B ratios have been observed during active disease which tend to increase after effective treatment, implying that a low F/B ratio may promote or reflect heightened mucosal and systemic inflammation [33,34]. In contrast, elevated F/B ratios have been reported in obesity [32], type 2 diabetes, and COVID-19 [35] cases, where they are often associated with metabolic and infection-related inflammation; this indicates that both abnormally low and high F/B ratios can accompany inflammatory pathology, depending on the clinical context.

In our study, a lower F/B ratio was observed in the vitamin D_3_-treated gut. Several reports indicate that vitamin D_3_ can influence gut microbiota by reshaping the relative abundances of Firmicutes and Bacteroidota. Although the mechanisms remain incompletely understood, higher circulating vitamin D_3_ levels have been linked to reduced F/B ratios and a gut microbiota profile that is more favorable for host health [36,37]. Alterations in the gut F/B ratio have been implicated in multiple inflammatory ocular diseases, including Sjögren’s syndrome [38], age-related macular degeneration (AMD) [39], and uveitis [40]. In AMD, a high-fat diet and complement activation—particularly subretinal deposition of C3 and C5—have been associated with an increased F/B ratio, and Bacteroidota have been proposed to exert a protective role in disease development [41,42,43]. Previously, our team demonstrated that inhibition of C3 and C5 attenuates myopia progression, further supporting a link between complement-mediated inflammation and ocular pathology [44]. Collectively, these findings suggest a potential positive association between ocular inflammation and the F/B ratio in the gut. They also raise the possibility that the myopia-suppressive effects of vitamin D_3_ are at least partly mediated through modulation of the F/B ratio and related inflammatory pathways in the gut–eye axis.

Both clinical studies and animal experiments indicate that although there is a significant difference in microbial diversity between the control and high myopia groups, their microbial compositions differ [13,45]. However, the present study detected no significant differences. This can possibly be attributed to our animal model, where we administered a small dose of the inflammatory cytokine via weekly eyelid injections and conducted the experiment for only 3 weeks, which was sufficient to induce myopia but not to cause high myopia. The vitamin D_3_ and TGF-β2 vitamin D_3_ group showed significantly different microbial abundance levels after 3 weeks of supplementation. Interestingly, Cyanobacteria, Vampirivibrionia, and Gastranaerophilales were enriched in the vitamin D_3_ group. Although they are less-studied gut residents and their specific immunomodulatory role in the gut remains unclear, emerging evidence suggests that cyanobacterial metabolites can affect immune and inflammatory pathways via short-chain fatty acids (SCFAs) [46].

Consistent with previous studies, our animal model also showed that vitamin D_3_ increases *Muribaculaceae* and *Bacteroides* abundance [47,48]. *Muribaculaceae*, many *Bacteroides*, and the *[Eubacterium]_xylanophilum_group* are known to play an anti-inflammatory role in the gut, and are related to SCFA production [26,49,50]. *Bacteroides* are related to myopia development; however, there is no direct evidence. A cross-sectional human study found that overall *Bacteroides* abundance may be higher in myopes [51]. However, at least one species (*Bacteroides intestinalis A*) appears to be causally protective in a Mendelian Randomization analysis [52]. Our results showed that *Bacteroides* is significantly negatively related to the elongation of axial length. These findings indicate that *Bacteroides* is indeed associated with the regulation of myopia and may be involved in the modulation of the F/B ratio by vitamin D_3_; however, this represents a complex mechanism that requires further investigation.

Another genus of interest, *Turicibacter*, exhibited a significant positive correlation with axial length. Certain strains within this genus have been reported to differentially modify bile acids, which possess well-recognized immunomodulatory properties [53]. Consistent with this concept, predicted functional profiling in our study indicated that metabolic pathways accounted for the majority of gut microbial functions; the four experimental groups displayed distinct metabolic signatures, including enrichment of bile secretion–related KEGG pathways.

The vitamin D receptor, which is activated by vitamin D_3_, has recently been shown to be a receptor for secondary bile acids that play a central role in calcium regulation, immune modulation, and bile acid metabolism [54,55]. In our study, we observed that hepatic CYP27A1—a mitochondrial sterol 27-hydroxylase that also serves as a minor vitamin D_3_ 25-hydroxylase contributing to local and systemic 25(OH)D_3_ production—was expressed at higher levels in the vitamin D_3_ and TGFβ2+vitamin D_3_ groups than in the control and TGFβ2 groups. In parallel, retinal VDR expression was significantly increased following vitamin D_3_ supplementation (Supplementary Figure S2). Activation of VDR by vitamin D_3_ or its analogs induces transcriptional changes in genes governing bile acid synthesis and transport, leading to significant alterations in bile acid composition in both the liver and circulation. This modulation can impact the metabolic and signaling roles of various bile acids, influencing host responses to inflammation and metabolic stress [21,56]. For instance, vitamin D_3_ and lithocholic acid, a secondary bile acid, interact with the VDRs that have protective effects against colon cancer and inflammatory bowel disease by attenuating inflammation and immune activation [57,58]. These results suggest that vitamin D_3_ may modulate microbiota-derived bile acids via VDR activation, thereby influencing bile acid metabolism and reshaping gut microbiota composition.

In this context, the modulation of vitamin D_3_ on microbiota-derived bile acids may, via VDR, extend to regulating the gut microbiota composition itself, with implications for gut, immune, and metabolic health. While there is no direct evidence connecting the specific bile acid derivatives examined in this study to myopia development, other bile acids—such as ursodeoxycholic acid (UDCA) and tauroursodeoxycholic acid (TUDCA)—exhibit anti-apoptotic, anti-inflammatory, and antioxidant activities in multiple models of neurodegenerative and retinal disease [59,60]. In the present work, 7-ketodeoxycholate levels were negatively associated with the genera *Bacteroides* and *Muribaculaceae*, both of which showed significantly increased abundance after vitamin D_3_ treatment compared to the control and TGF-β2 groups (Supplementary Figure S3). These datapoints indicate that bile acids are important immune regulators, and the observed associations between bile acid derivatives, axial elongation, and refractive changes parallel the pattern seen for Turicibacter in our model. This convergence raises the possibility that gut microbiota–derived bile acids, potentially modulated by vitamin D_3_ through alterations in microbial community structure, contribute to the suppression of retinal inflammation and thereby help restrain myopia progression.

Imidazole and its derivatives have shown significant potential in the treatment of ocular diseases due to their anti-inflammatory properties [61,62]. Here, we showed that ImP and MIAA increased following vitamin D_3_ administration and were significantly negatively related to axial length. In summary, we demonstrated a direct association between vitamin D_3_ and the imidazole derivatives ImP and MIAA, proposing that they may serve as potential therapeutic targets for myopia.

In the present study, no statistically significant changes in serum 25(OH)D_3_ concentrations were observed at baseline or post-supplementation, either within individual groups or between groups (Supplementary Table S3). Prior studies support the notion that sustained supplementation at comparable doses is sufficient to modify vitamin D status in rats. In 6-week-old Sprague–Dawley rats, 6 weeks of 1000 IU/kg vitamin D_3_ elevated serum 25(OH)D_3_ relative to unsupplemented controls [63], and a linear dose–response relationship in 25(OH)D_3_ was demonstrated following 4 weeks of dietary vitamin D_3_ ranging from 400 to 20,000 IU/kg [64].

Nevertheless, several studies indicate that vitamin D_3_ supplementation does not necessarily increase circulating 25(OH)D_3_ concentrations. In one study, 7-week-old Sprague–Dawley rats were rendered vitamin D_3_-deficient by consuming an AIN-93M diet for 10 days and then received 1000 IU/kg vitamin D_3_; plasma 25(OH)D_3_ levels after 4 weeks of supplementation did not differ significantly from either the Day 0 baseline or the Day 10 deficiency level [65]. Similarly, 8-week-old CD-1 mice administered 10,000 IU/kg vitamin D_3_ as either a single dose or once daily for one week showed no significant change in serum 25(OH)D compared with control mice maintained on regular chow without vitamin D_3_ supplementation [66]. Consistent with this notion, the absence of a statistically significant increase in serum 25(OH)D_3_ in our study is likely related to the relatively short 21-day supplementation period rather than a lack of biological activity. This interpretation is supported by our observations of attenuated ocular inflammation and shifts in gut microbiota composition, indicating local tissue-level responses to vitamin D_3_ despite only modest changes in systemic metabolite levels.

There are certain limitations to our study. First, the animal model used did not mimic high myopia, which may partially account for the absence of significant differences in microbial and metabolic profiles between the myopia and control groups. Second, the present work specifically examined bile acids, imidazole derivatives, and the inhibitory effects of vitamin D_3_ on myopia; however, it did not include an assessment of short-chain fatty acids (SCFAs). SCFAs, such as butyrate, are generated by gut bacteria through the fermentation of dietary fiber. They have been shown to enhance vitamin D receptor (VDR) signaling, which is critical for the immunomodulatory actions of vitamin D_3_ [67,68]. Notably, butyrate strengthens gut barrier integrity and modulates immune responses in a manner that parallels the effects of vitamin D_3_. Future studies incorporating SCFA profiling will be important to further elucidate how vitamin D_3_-mediated myopia suppression interacts with gut microbiota composition and function.

## 5. Conclusions

Our study demonstrates that vitamin D_3_ modulates the gut microbial ecosystem by altering the Firmicutes-to-Bacteroidota ratio and reshaping specific bacterial taxa, while increasing the levels of the microbial metabolites imidazolepropionic and methylimidazoleacetic acid. In addition to attenuating myopia progression, these alterations support the involvement of a gut–eye axis in the protective effects of vitamin D_3_ on myopia development. Targeting these vitamin D_3_-responsive taxa and metabolites may therefore represent a promising strategy for preventing or slowing myopia progression.

## Figures and Tables

**Table 1:** Alpha-diversity indices (Chao 1 index and Shannon index) of cecal microbiota in control, vitamin D_3_, TGF-β2, and TGF-β2+vitamin D_3_ groups.

Index	Group 1	Group 2	Mean 1	Mean 2	Std 1	Std 2	Statistic	*p*-Value
**Chao 1**	Control	TGF-β2	551.342	523.361	29.568	32.457	1.692	0.114
	Control	Vitamin D_3_	551.342	530.559	29.568	17.104	1.735	0.106
	Control	TGF-β2+Vitamin D_3_	551.342	517.069	29.568	32.405	2.075	0.058
	TGF-β2	Vitamin D_3_	523.361	530.559	32.457	17.104	−0.589	0.564
	TGF-β2	TGF-β2+Vitamin D_3_	523.361	517.069	32.457	32.405	0.412	0.686
	Vitamin D_3_	TGF-β2+Vitamin D_3_	530.559	517.069	17.104	32.405	1.105	0.286
**Shannon**	Control	TGF-β2	5.746	5.512	0.521	0.693	0.704	0.494
	Control	Vitamin D_3_	5.746	5.684	0.521	0.381	0.267	0.794
	Control	TGF-β2+Vitamin D_3_	5.746	5.815	0.521	0.347	−0.311	0.761
	TGF-β2	Vitamin D_3_	5.512	5.684	0.693	0.381	−0.654	0.522
	TGF-β2	TGF-β2+Vitamin D_3_	5.512	5.815	0.693	0.347	−1.176	0.257
	Vitamin D_3_	TGF-β2+Vitamin D_3_	5.684	5.815	0.381	0.347	−0.764	0.456

**Table 2:** Beta-diversity metrics and PERMANOVA statistics for cecal microbiota among control, vitamin D_3_, TGF-β2, and TGF-β2+vitamin D_3_ groups.

Comparison	F (Pseudo-F)	R^2^	*p*-Value
**Control vs. TGF-β2**	−8.564	−1.931	0.471
**Control vs. Vitamin D_3_**	−8.936	−2.199	0.032 *
**Control vs. TGF-β2+Vitamin D_3_**	−8.342	−1.791	0.013 *
**TGF-β2 vs. Vitamin D_3_**	−10.949	−2.168	0.032 *
**TGF-β2 vs. TGF-β2+Vitamin D_3_**	−10.192	−1.755	0.007 *
**Vitamin D_3_ vs. TGF-β2+Vitamin D_3_**	−12.016	−3.016	0.533

Statistical significance: * *p* < 0.05.

## Data Availability

All data generated or analyzed during this study are included in this published article and its Supplementary Materials.

## Supplementary Materials

The following supporting information can be downloaded at: https://www.mdpi.com/article/10.3390/biom16070939/s1, Figure S1: Vitamin D_3_ suppressed myopia-specific markers in the retina. Figure S2: Vitamin D_3_ activated hepatic CYP27A1 and VDR in the retina. Figure S3: Associations between vitamin D_3_-responsive genera and microbiota-derived metabolites. Table S1: Relative levels of gut microbiota–derived bile acids and imidazole derivatives in TGF-β2, vitamin D_3_, and TGF-β2+vitamin D_3_ groups compared with the control group. Table S2: Relative levels of gut microbiota–derived bile acids and imidazole derivatives in control, vitamin D_3_, and TGF-β2+vitamin D_3_ groups compared with the TGF-β2 group. Table S3: Statistical comparison of serum 25(OH)D concentrations on Day 21 versus Day 1 in the four experimental groups.

## Abbreviations

The following abbreviations are used in this manuscript:

3-IAA	Indole-3-acetic acid
VDR	Vitamin D receptor
FDM	Form-deprivation myopia
PCoA	Principal Coordinates Analysis
NMDS	Non-metric multidimensional scaling
LEfSe	Linear Discriminant Analysis Effect Size
STAMP	Statistical Analysis of Metagenomic Profiles
PICRUSt	Phylogenetic Investigation of Communities by Reconstruction of Unobserved States
PERMANOVA	Permutational multivariate analysis of variance
FDR	False discovery rate
F/B	Firmicutes-to-Bacteroidota ratio
KEGG	Kyoto Encyclopedia of Genes and Genomes
ImP	Imidazolepropionic acid
MIAA	Methylimidazoleacetic acid
HDCA	Hyodeoxycholic acid
DCA	Deoxycholic acid
GCA	Glycocholic acid
AMD	Age-related macular degeneration
SCFAs	Short-chain fatty acids
UDCA	Ursodeoxycholic acid
TUDCA	Tauroursodeoxycholic acid
