# Supplementary material for: Vitamin D3 Reshapes Gut Microbiota and Metabolite Profiles in a Rat Model of Inflammation-Induced Myopia
Source: Biomolecules. 2026 Jun 24;16(7):939. doi: 10.3390/biom16070939 (PMC13406523; doi:10.3390/biom16070939)
Supplement: Supplementary file 1 [file biomolecules-16-00939-s001.zip › biomolecules-4220680-supplementary.pdf]

Table S1. Relative levels of gut microbiota–derived bile acids and imidazole derivatives in TGF-β2, vitamin D_3, and TGF-β2+vitamin D_3 groups compared with the control group.

Compounds	TGF-β2			Vitamin D_3			TGF-β2+ Vitamin D_3		
	z-score	Log2FC	p-value	z-score	Log2FC	p-value	z-score	Log2FC	p-value
Imidazolepropionic acid	0.541	0.286	0.567	3.319	1.648	**0.006***	1.518	1.263	0.168
Methylimidazoleacetic acid	0.260	0.099	0.770	2.959	1.644	**0.011***	1.391	1.104	0.211
Deoxycholic acid	0.060	0.085	0.946	-1.365	-1.243	0.256	-1.581	-1.709	0.194
Hyodeoxycholic acid	0.158	0.210	0.823	-0.762	-2.050	0.323	-0.683	-1.737	0.366
Glycocholic acid	0.137	0.171	0.875	-0.911	-1.446	0.370	-1.246	-2.719	0.262
7-Ketodeoxycholate	0.023	0.035	0.979	-0.809	-1.064	0.419	-1.324	-2.765	0.237

Table S2. Relative levels of gut microbiota–derived bile acids and imidazole derivatives in control, vitamin D_3, and TGF-β2+vitamin D_3 groups compared with the TGF-β2 group.

**Compounds**	**Control**			**Vitamin D_3**			**TGF-β2+ Vitamin D_3**		
	**z-score**	**Log2FC**	**p-value**	**z-score**	**Log2FC**	**p-value**	**z-score**	**Log2FC**	**p-value**
Imidazolepropionic acid	-0.541	-0.286	0.567	3.542	1.363	**0.008***	1.503	0.978	0.225
Methylimidazoleacetic acid	-0.260	-0.099	0.770	2.951	1.545	**0.016***	1.344	1.005	0.235
Deoxycholic acid	-0.060	-0.085	0.946	-0.784	-1.328	0.477	-0.918	-1.794	0.412
Hyodeoxycholic acid	-0.158	-0.210	0.823	-10.424	-2.260	**0.001***	-2.979	-1.948	0.054
Glycocholic acid	-0.137	-0.171	0.875	-1.176	-1.617	0.271	-1.547	-2.891	0.193
7-Ketodeoxycholate	-0.023	-0.035	0.979	-0.637	-1.098	0.514	-1.024	-2.800	0.332

Statistical significance: * $p < 0.05$.

Table S3. Statistical comparison of serum 25(OH)D concentrations on Day 21 versus Day 1 in the four experimental groups.

Group	p-value	Mean of Day 1	Mean of Day 2	Difference between means ± SEM	95 confidence interval	R squared
**Control**	0.7068	16.47	15.77	-0.7030 ± 1.740	-5.533 to 4.127	0.03922
**Vitamin D_3**	0.5020	11.66	10.83	-0.8257 ± 1.120	-3.936 to 2.285	0.1196
**TGF-β2**	0.5017	18.26	15.13	-3.128 ± 4.240	-14.90 to 8.646	0.1197
**TGF-β2 + Vitamin D_3**	0.4170	13.85	12.15	-1.696 ± 1.875	-6.902 to 3.510	0.1697

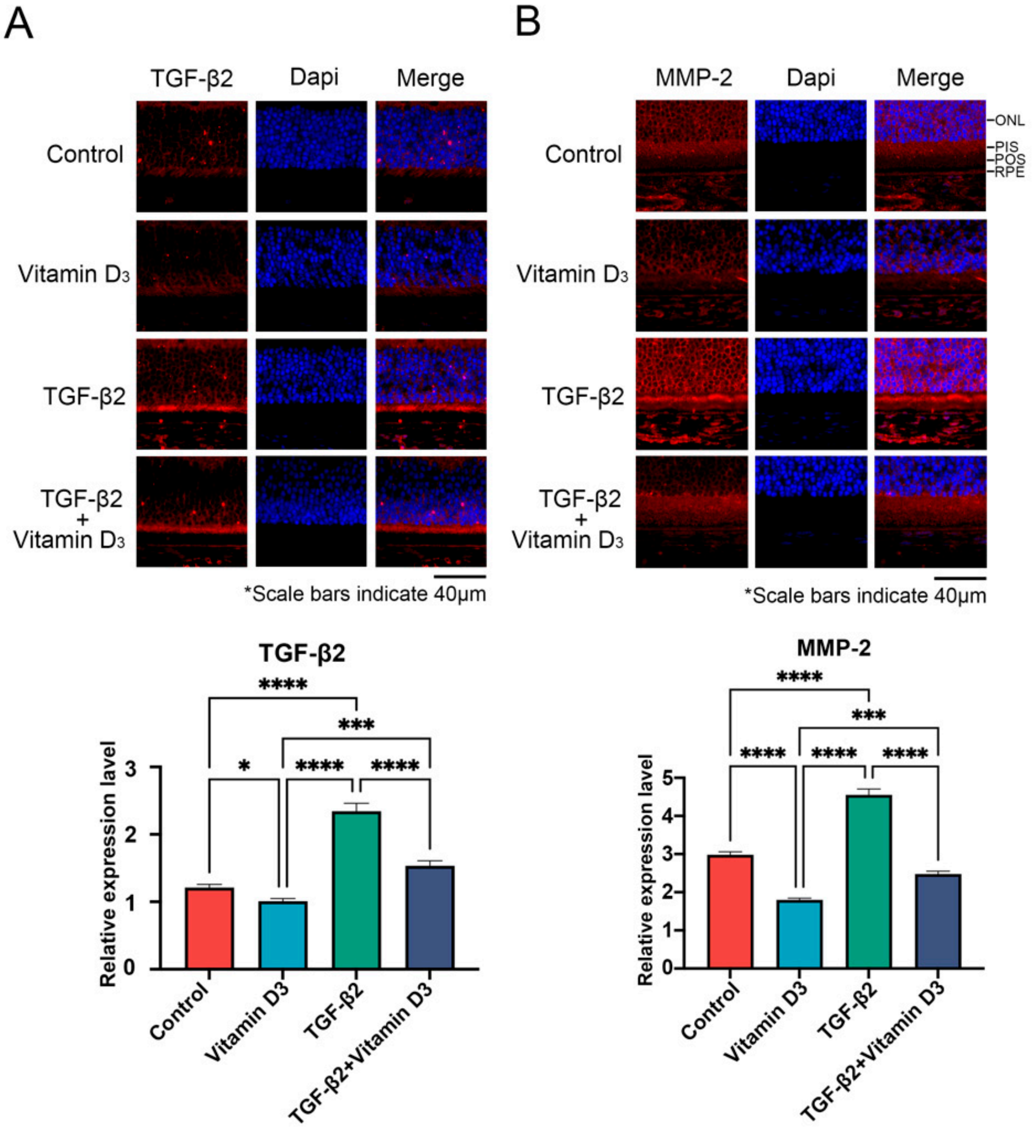

Figure S1. Vitamin D_3 suppressed myopia-specific markers in the retina.

(A, B) Immunofluorescence staining shows retinal expression of the myopia-specific markers TGF-β2 (A) and MMP-2 (B) in control, vitamin D_3, TGF-β2, and TGF-β2 + vitamin D_3 groups, with relative fluorescence intensities shown below. Differences among groups were evaluated using one-way ANOVA followed by post hoc multiple comparisons. Statistical significance: * $p < 0.05$, ** $p < 0.01$, *** $p < 0.0005$, **** $p < 0.0001$, ns: not significant. ONL, outer nuclear layer; PIS, photoreceptor inner segments; POS, photoreceptor outer segments; RPE, retinal pigment epithelium.

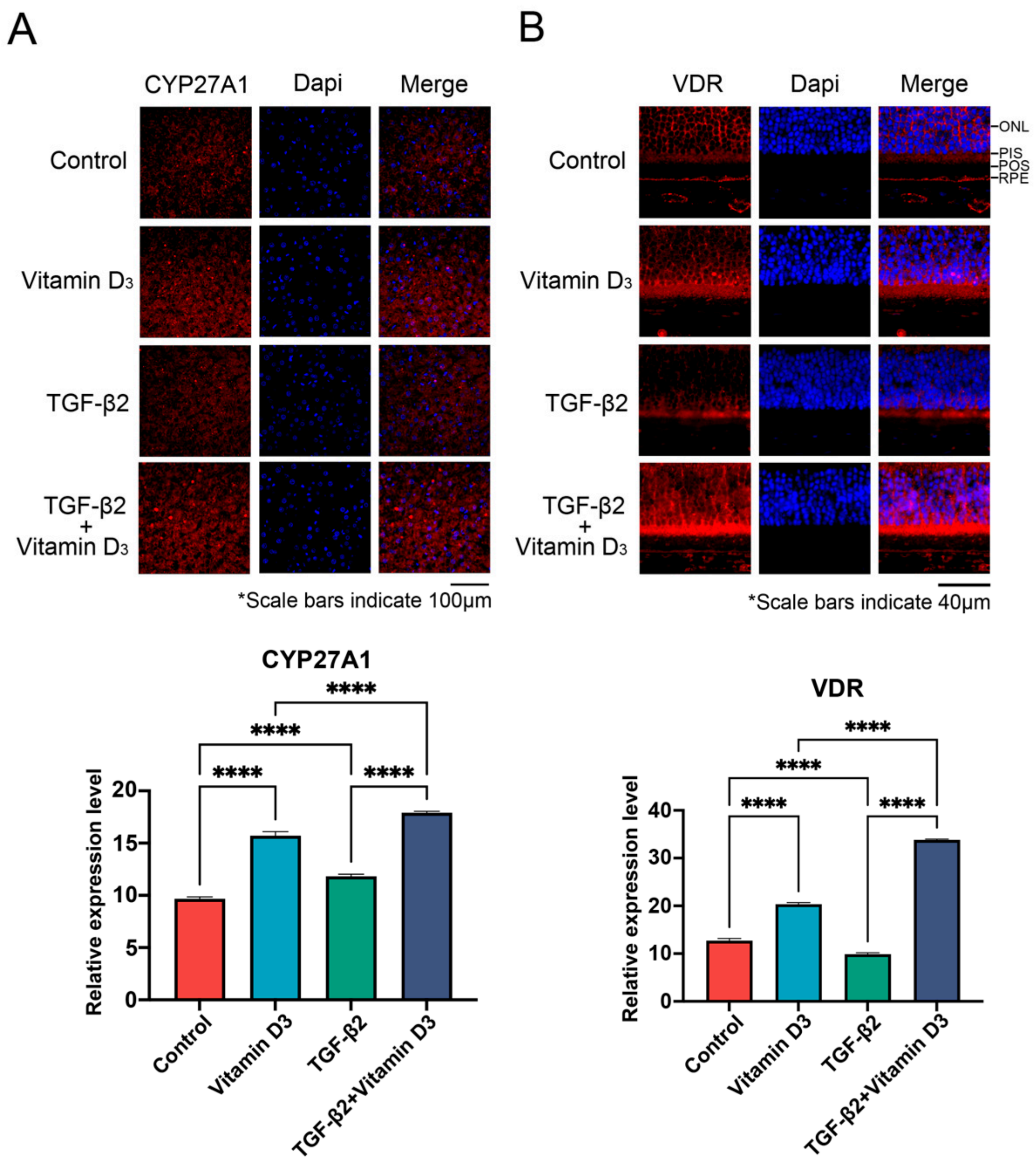

Figure S2. Vitamin D3 enhances vitamin D3 metabolic activation in the liver and retina.

(A) Immunofluorescence staining of liver sections showing CYP27A1 expression in each treatment group. (B) Immunofluorescence staining of retinal sections showing VDR expression in control, vitamin D_3, TGF-β2, and TGF-β2 + vitamin D_3 groups, with relative fluorescence intensities shown below. Differences among groups were evaluated using one-way ANOVA followed by post hoc multiple comparisons. Statistical significance: **** p <0.0001, ns: not significant. ONL, outer nuclear layer; PIS, photoreceptor inner segments; POS, photoreceptor outer segments; RPE, retinal pigment epithelium.

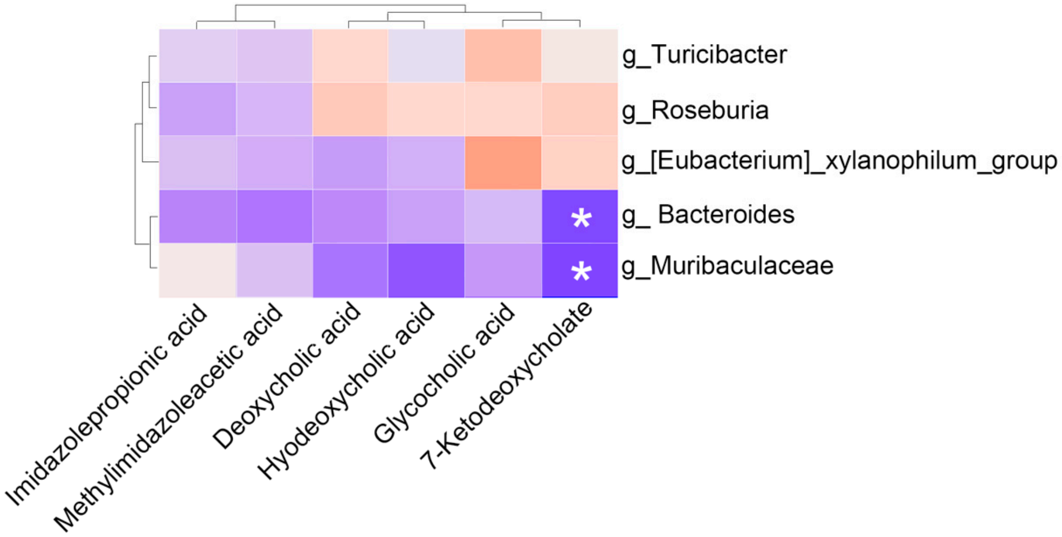

Figure S3. Associations between vitamin D_3-responsive genera and microbiota-derived metabolites.

Spearman's correlation analysis between differential bacteria identified from STAMP analysis and metabolites. * p <0.05 indicates statistical significance.
